# Cellular characteristics of the immune microenvironment of colorectal cancer and progress in immunotherapy research

**DOI:** 10.1080/07853890.2025.2591308

**Published:** 2025-11-30

**Authors:** Chunbaixue Yang, Jianchun Fan, Yixuan Zhang, Yixiao Zhang, Lei Xia, Xinran Cao, Xueliang Wu

**Affiliations:** ^a^ Graduate School, Hebei North University, Zhangjiakou, China; ^b^ Institute of Oncology, The First Affiliated Hospital of Hebei North University, Zhangjiakou, China; ^c^ Dept of General Surgery, The First Affiliated Hospital of Hebei North University, Zhangjiakou, China; ^d^ The First Clinical Medical School, Hebei North University, Zhangjiakou, China; ^e^ Clinical Laboratory, The First Affiliated Hospital of Hebei North University, Zhangjiakou, China

**Keywords:** Colorectal cancer, immunotherapy, research progress, tumor microenvironment

## Abstract

**Background:**

Colorectal cancer (CRC) continues to represent a major cause of cancer-related mortality worldwide, with its progression and therapeutic outcomes strongly shaped by the complexity and heterogeneity of the tumor immune microenvironment (TME). This review critically examines the cellular and molecular mechanisms driving immune evasion in CRC, emphasizing the dual roles of immune cell populations—including tumor-associated macrophages, neutrophils, dendritic cells, T cells, B cells, and natural killer cells—as well as non-cellular elements such as the extracellular matrix and extracellular vesicles.

**Objective:**

A key objective is to evaluate recent developments in immunotherapeutic approaches, including immune checkpoint inhibitors, tumor vaccines, adoptive cell transfer, and novel combinatorial regimens, while addressing their therapeutic promise and inherent limitations, especially in microsatellite-stable (MSS) tumors that exhibit primary resistance to standard immunotherapies. Further analysis integrates perspectives on metabolic reprogramming within the TME, epigenetic alterations, and advances in engineered cellular therapies, thereby providing a comprehensive framework for overcoming immunosuppressive mechanisms.

**Discussion and conclusion:**

Special consideration is directed toward the translational value of targeting immune-metabolic interactions and spatial dynamics within the TME. Ultimately, this work synthesizes current knowledge and outlines forward-looking strategies to advance personalized, multi-target immunotherapy, with the potential to reshape clinical paradigms in CRC management.

## 1. Introduction

According to recent epidemiological statistics, colorectal cancer (CRC) is the third most common malignancy after lung and breast cancer and ranks second cancer-related mortality, reflecting the unfavorable prospects for prevention and treatment efforts [1]. Against this backdrop, comprehensive investigation of CRC pathogenesis and the development of more effective therapeutic strategies remain imperative. Accumulating evidence highlights the complex association between CRC initiation and progression and the tumor microenvironment (TME) [2]. Dynamic alterations within the TME substantially influence disease trajectory and therapeutic responsiveness. The TME refers to the cellular and molecular milieu surrounding the tumor or cancer stem cells and includes diverse cellular populations, cytokines, and structural components such as the extracellular matrix, CD4^+^ T cells, CD8^+^ T cells, B cells, and M1-type or N1-type neutrophils act to suppress CRC progression, whereas M2-type tumor-associated macrophages and N2-type tumor-associated neutrophils promote immune evasion and accelerate disease development [3]. Despite advances, the mechanistic roles of the CRC TME remain insufficiently defined and warrant deeper investigation. Designing therapeutic protocols that specifically target the TME has emerged as a major focus of current research. Within the contemporary therapeutic landscape, immunotherapy has become a key approach complementing conventional surgery, chemotherapy, radiotherapy, and targeted therapy. Although certain immunotherapies demonstrate measurable benefits, resistance and heterogeneous patient responses limit their efficacy. Functional and phenotypic analyses of immune cells within the CRC tumor immune microenvironment have provided insights into therapeutic potential and clinical outcomes. Current research trends in immunotherapy are systematically summarized to establish a theoretical foundation for optimizing treatment strategies for CRC.

## 2. Cellular phenotypes and mechanisms of action of the colorectal cancer tumor immune microenvironment

### 2.1. Tumor-associated dendritic cells

Although certain immunotherapies demonstrate measurable benefits, resistance and heterogeneous patient responses limit their efficacy. Functional and phenotypic analyses of immune cells within the CRC tumor immune microenvironment have provided insights into therapeutic potential and clinical outcomes. Current research trends in immunotherapy are systematically summarized to establish a theoretical foundation for optimizing treatment strategies for CRC [4,5]. Functioning as professional antigen-presenting cells DCs mediate tumor surveillance by regulating B-cell proliferation and differentiation, delivering tumor antigens to T cells, and initiating cytotoxic T-cell-driven elimination of tumor cells [6,7]. Their maturation of DCs within the TME is primarily stimulated by by interferon (IFN) I in the tumor microenvironment (TME) [8], whereas factors such as vascular endothelial growth factor (VEGF) and transforming growth factor (TGF)-β impede this process and attenuate DC function [9]. In MC38 CRC mice, regulatory dendritic cells (DCregs), particularly BATF3^+^ type 1 conventional DCs (DC1s), were shown to induce T-cell unresponsiveness through CXCL9 production, which recruits CXCR3^+^ regulatory T cells (Tregs) into the TME. These CXCR3^+^ Tregs display an activated phenotype and preferentially engage with DC1s, thereby diminishing their antigen cross-presentation and weakening CD8^+^ T-cell priming and reactivation. Moreover, PD-L1 expression on regulatory DCs further suppresses T-cell proliferation, reinforcing immune tolerance and promoting tumor progression. Interfering with the CXCR3–CXCL9 axis may disrupt this immunosuppressive network and restore antitumor immunity [10].

Relevant investigations into the multifaceted role of DCs in CRC are expected to yield insights for immunotherapeutic strategies. Yuan et al. [11] identified a positive association between the expression of chemokine C-X-C motif ligand (CXCL) family members and DC-related markers. Chemokines such as CCL19, CCL21, and XCL1 increased the proportions of DCs and T cells, while CCL3 enhanced the frequency of CD45^+^ leukocytes. Overexpression of XCL1 promoted antigen by DCs, expanded the pool of classical DCs, and strengthen the antitumor immune responses within the CRC TME. Upregulation of CXCL8 correlated coincided with increased expression of genes implicated in DC activation pathways (e.g., CD80, CD83, and CD86). Inhibition of the CXCL8-CXCR1/2 axis impeded DC activation or recruitment, a process considered to reinforce antitumor responses in CRC [12]. In addition, glycosylated tumor antigens such as CEA and MUC1 on CRC cells have been shown to serve as ligands for DC-SIGN (CD209) on DCs. Engagement of DC-SIGN by tumor-derived ligands suppresses DC maturation by reducing co-stimulatory molecule expression (e.g., CD80, CD86) and limiting pro-inflammatory cytokine production, thereby weakening the initiation of effective T-cell responses. Activation of DC-SIGN by fucose-containing glycans (e.g., Lewis antigens) induces IL-10 expression and drives Th2 differentiation, creating an immunosuppressive microenvironment. Clinically, the presence of DC-SIGN+ macrophages correlates with immune suppression and adverse outcomes in gastric cancers, directly linking this mechanism to tumor progression and metastasis. Such interactions compromise DC function, weaken antitumor T-cell priming, and reinforce an immunosuppressive milieu that supports immune evasion in CRC [13–15].

### 2.2. Tumor-associated macrophages

CCR2^+^ monocytes in the bone marrow constitute the major precursor pool for tumor-associated macrophages (TAMs), and their differentiation into this specialized population is markedly intensified during tumor progression. The accumulation of TAMs within the TME is largely governed by the recruitment of circulating monocytes. Functionally, TAMs are broadly divided into two phenotypes: the proinflammatory M1 type and the alternatively activated M2 type. M1 macrophages exert antitumor activity by generating reactive oxygen species (ROS) and secreting inflammatory mediators such as interferon-gamma (IFN-γ). In contrast, M2-polarized TAMs predominantly release interleukins including IL-4, IL-10, and IL-13, thereby suppressing T cell–mediated immune responses and promoting angiogenesis within tumors. Together, these processes sustain malignancy by reinforcing immune evasion and augmenting nutrient delivery to neoplastic tissue. Among the regulatory mechanisms, the CSF1 signaling axis plays a decisive role in shaping TAM polarization. Engagement of CSF1 with its receptor CSF1R drives the transition toward an M2-like phenotype, which dampens T-cell effector functions and accelerates tumor advancement [16–21].

TGF-β, prostaglandin E2, and various cytokines secreted by TAMs suppress T-cell activity and induce PD-L1 expression. PD-L1, expressed on CRC tumor cell surfaces, binds to programmed cell death protein 1 (PD-1) on T cells, diminishing cytotoxic function and promoting immune evasion. A high density of TAMs has been consistently linked to resistance against PD-1/PD-L1 immunotherapies [22].

Single-cell RNA sequencing revealed that SPP1 was abundantly expressed in a TAM subset characterized by a senescence-associated secretory phenotype. In highly malignant CRC, Yu et al. [23] demonstrated that SPP1^+^ TAMs were surrounded by numerous senescent tumor cells, indicating their association with cellular senescence and unfavorable prognosis. Elevated expression of inhibitor of differentiation 1 (ID1) in CRC also correlates with poor outcomes. ID1 binds signal transducer and activator of transcription 1 (STAT1), alters its cytoplasmic localization, and represses transcription of STAT1-regulated downstream targets, including SerpinB2 and CCL4. Moreover, ID1+ TAMs sustain tumor stemness and restrict CD8^+^ T-cell infiltration in CRC, thereby representing a potential therapeutic target [24]. Nicotinamide phosphoribosyltransferase (NAMPT), a metabolic enzyme with proinflammatory roles, is implicated in TAM activation and CRC progression. Conditional deletion of macrophage-specific NAMPT delayed CRC development in a murine model. Subsequent studies identified NAMPT as a key regulator of TAM activation, mediating HIF-1α/STAT3 signaling and suppressing STING pathway rearrangement. Loss of NAMPT in TAMs reduces M2 polarization, enhances STING signaling and type I interferon responses, and enhances cytotoxic T-cell activity, collectively reinforcing antitumor immunity [25].

### 2.3. Tumor-associated neutrophils

Tumor-associated neutrophils (TANs), originating from myeloid progenitors, are divided into N1 and N2 subsets. In the early phase of tumor development, the cytotoxic N1 subtype predominates, but with tumor progression, a phenotypic shift toward the N2 subtype occurs. N1 TANs restrict tumor growth by enhancing IL-18 expression in natural killer (NK) cells through INF-1 secretion and by directly inducing tumor cell death via myeloperoxidase, ROS, and related mediators. Exposure to TGF-β subsequently drives the conversion of N1 cells into the N2 phenotype. N2 TANs contribute to CRC progression through mechanisms including ROS generation, inhibition of CD8^+^ T cell activity, suppression of NK cell function, and promotion of tumor cell extravasation [26,27]. Beyond direct cytotoxic or protumorigenic effects on tumor cells, TANs engage in intricate crosstalk with other immune cell populations, particularly TAMs. Within the inflammatory microenvironment, TANs together with TAMs foster tumor progression and metastatic dissemination [28].

CD66b, a 95–100 kDa glycosylphosphatidylinositol-linked protein, is abundantly expressed on neutrophils and serves as a reliable marker for identifying TANs in CRCs [29]. Elevated infiltration of TANs has been observed both at invasive margins and within central tumor regions. Notably, TNF-α–positive TANs accumulate at tumor boundaries, exhibit antitumor activity, and display higher expression of intercellular adhesion molecule-1 and CD95 compared with neutrophils in normal tissues, indicating their potential as prognostic indicators for CRC patients [30]. Neutrophil extracellular traps (NETs), reticular structures generated through NETosis, consist of DNA filaments and cytotoxic enzymes released into the extracellular milieu. In murine models, IFNγ treatment enhances NET formation and apoptosis, reduces tumor burden, and strengthens PD-1 antibody–mediated tumor cytotoxicity in MSS CRC cell lines [31]. During neutrophil trafficking from bone marrow to peripheral tissues, aryl hydrocarbon receptor nuclear translocator (ARNT) deficiency promotes neutrophil recruitment, NET release, proinflammatory cytokine secretion, and immunosuppressive activity, thereby driving colorectal tumorigenesis. CXCR2 is markedly upregulated in neutrophils from ARNT-deficient mice, increasing their migratory and functional capacities. Inhibition of CXCR2 diminishes neutrophil recruitment, restrains NET formation, and reduces both incidence and progression of CRC [32]. In advanced CRC, TANs express Spp1 (OPN) and Mmp14 (MT1-MMP), with angiogenic TANs exhibiting particularly high levels of these proteins. Through OPN and MMP14 release, TANs enhance endothelial cell migration and stimulate spontaneous vascular sprouting and branching, collectively accelerating CRC progression [33].

### 2.4. T cells

T cells are intimately linked to tumor progression and immune suppression. The surface T-cell receptor (TCR) functions as a central signaling complex by recognizing major histocompatibility complex (MHC) molecules and initiating intracellular cascades that drive activation, proliferation, and differentiation of T cells into effector subsets capable of eliminating malignant cells [34]. Tumor-associated antigens provoke a tumor-specific immune response, leading to activation of MHC class I-restricted CD8^+^ T cells and MHC class II-restricted CD4^+^ T cells, with the latter exerting essential regulatory and effector roles in antitumor immunity [35].

#### 2.4.1. CD8^+^ T cells

CD8^+^ cytotoxic T lymphocytes eradicate tumor cells through two cooperative mechanisms: the perforin/granzyme pathway induces rapid lysis of target cells, while secretion of cytokines such as IL-2, IFN-γ, and TNF-α establishes a proinflammatory milieu that sustains long-term cytotoxic activity [36]. In early-stage CRC, infiltration of activated CD8^+^ T cells into peritumoral and precancerous tissues correlates with improved prognosis, whereas patients with stage I or II CRC who lack CD8^+^ T-cell infiltration face a markedly elevated risk of recurrence within 5 years [37].

CRC cells reshape the metabolic profile of CD8+ T cells by employing regulatory mechanisms such as excessive lactate secretion. Lactate diminishes cytotoxic activity by shifting T-cell metabolism from pyruvate carboxylase toward pyruvate dehydrogenase [38]. Exosomal miR-1246 further contributes to immune evasion by reducing CCL2 expression, thereby limiting CD8+ T-cell recruitment to tumor sites and diminishing antitumor responses [39].

Beyond the perforin/granzyme axis and cytokine-mediated mechanisms, the functional diversity of CD8+ T-cell subsets within the CRC microenvironment has emerged as a determinant of clinical outcome. Tissue-resident CD103^+^CD8^+^ T cells have been linked to longer survival and lower incidence of distant metastasis, representing an independent prognostic indicator [40]. In contrast, TIGIT expression on CD8^+^ T cells induces an exhausted phenotype marked by reduced IFN-γ, IL-2, and TNF-α secretion together with upregulation of inhibitory receptors (PD-1, TIM-3, Lymphocyte activation gene-3 (LAG-3)), a pattern associated with disease progression and unfavorable prognosis [41]. Neutrophil infiltration (CD66b^+^) has been shown to enhance CD8^+^ T-cell activation, proliferation, and memory differentiation via direct CD11a/CD54 interactions, and concurrent infiltration of neutrophils and CD8^+^ T cells markedly improves patient survival [42].

#### 2.4.2. CD4^+^ T cells

Within the TME, CD4^+^ T cells demonstrate marked functional plasticity through differentiation into Th1, Th2, or Treg subsets, exerting either inhibitory or promotive effects on tumor progression. Secretion of IL-2, IFN-γ, and TNF-α by these cells directly suppresses tumor growth while simultaneously enhancing the cytotoxic activities of other immune components [43,44]. By releasing cytokines such as IFN-γ, TNF, and IL-2, CD4^+^ T cells further promote the differentiation and activation of macrophages, CD8^+^ T cells, and NK cells, while limiting tumor-associated angiogenesis, thereby reinforcing antitumor responses [45].

Evidence indicates that chronic intestinal inflammation and aberrant immune activation are central drivers of CRC pathogenesis. Th1 cells, through IFN-γ secretion, mediate cell-dependent immunity and inflammatory processes, whereas Th2 cells release IL-4, IL-5, and IL-13, which foster B-cell maturation into antibody-producing cells and sustain humoral immunity. In CRC, alterations in CD4^+^ T-cell responses, including subset distribution and functional adaptability, reshape the equilibrium between tumor-promoting and tumor-suppressive immunity in accordance with the prevailing inflammatory context [46–48].

#### 2.4.3. Regulatory T cells

Tregs, defined as CD4^+^ T lymphocytes expressing CD25 and the transcription factor Foxp3 [49], engage dynamically with tumor cells and extracellular matrix components. Tumor-derived mediators, such as mid-stage cytokine (MDK), recruit Tregs and establish an immunosuppressive milieu. MDK binds to syndecan-4 (SDC4) on Tregs, enhancing their accumulation and suppressive activity during the early phases of CRC progression [50]. Additional chemokine signaling, particularly the CCL22–CCR4 axis, further augments Treg enrichment within the CRC TME [51].

The functional regulation of Tregs is governed by both intrinsic and extrinsic determinants. FoxP3 operates as the central transcriptional regulator of Treg lineage specification and suppressive capacity [52].Signals through TCR, CD28, and cytokine receptors such as IL-2R sustain Treg activation, stability, and proliferative potential [53]. In parallel, metabolites abundant in the TME, including adenosine and lactate, modulate the intensity and adaptability of Treg-mediated immunosuppression [54,55].

Tregs drive tumor progression within the TME through a spectrum of direct and indirect suppressive mechanisms. By expressing inhibitory receptors such as cytotoxic T lymphocyte-associated antigen-4 (CTLA-4) (which binds to CD80/86 derived from cancer cells) and PD-1, Tregs disrupt costimulatory signaling and suppress antitumor T-cell activity. They also impose metabolic constraints via the adenosine-generating CD39/CD73 ectoenzyme pathway and IL-2 depletion. In addition, Tregs can induce cytolysis of effector populations, including antigen-presenting cells and T effector cells, through granzyme B and perforin release [56–58].

Within the TME, suppression of CD8^+^ and CD4^+^ effector T cells is further reinforced by the secretion of inhibitory cytokines (IL-10, TGF-β), competitive IL-2 consumption, and adenosine production, collectively dampening antitumor immune responses [59,60]. Recent studies using CRC mouse models and human CRC samples have identified spatial interactions between Tregs and mature regulatory dendritic cells (mregDCs) with immunoregulatory features, predominantly localized around lymphatic vessels in the tumor stroma. These interactions intensify Treg activation and expand their suppressive capacity in the TME [61]. Moreover, TGF-β derived from Tregs activates the SMAD signaling pathway in NK cells, leading to reduced expression of the activating receptor NKG2D and diminished recognition and killing of tumor cells [62,63].

#### 2.4.4. The intrinsic metabolic pathways of T cells and TME

The hexosamine biosynthesis pathway (HBP) and the tricarboxylic acid (TCA) cycle represent central metabolic circuits governing T cell differentiation and activity, and their dysregulation within the CRC TMIE drives immunosuppressive outcomes.

The TCA cycle, as the principal hub of cellular energy metabolism and a source of biosynthetic precursors [64], exerts direct control over CD8^+^ T cell fate and functional capacity. Succinate, a key intermediate of this pathway, enhances CD8^+^ T cell antitumor responses by sustaining mitochondrial integrity through BNIP3-dependent mitophagy, thereby limiting mitochondrial ROS accumulation and apoptotic signaling [65]. Recent evidence indicates that succinate also reprograms the epigenetic landscape by modulating the succinate/α-ketoglutarate balance, enriching activating H3K4me3 while diminishing repressive H3K27me3 at stemness-related loci such as TCF-1, which preserves stem-like properties and long-term persistence of T cells. Tumor cells can exploit TCA cycle alterations to evade immune attack; for instance, DUSP4 expression in microsatellite-instable (MSI) CRC suppresses ferroptosis, an immunogenic form of cell death, thereby weakening T cell–mediated cytotoxic activity [66].

The HBP, a branch of glucose metabolism, produces UDP-GlcNAc for *O*-GlcNAcylation, a post-translational modification that modulates signaling proteins and transcription factors [67]. In cancer cells, enhanced glucose uptake and HBP flux increase UDP-GlcNAc availability, driving elevated *O*-GlcNAcylation and activating diverse pathways linked to oncogenesis and stress adaptation [68]. *O*-GlcNAc modification regulates the equilibrium between Th17 (pro-inflammatory) and Treg (anti-inflammatory) cells by influencing the stability and transcriptional activity of RORγt, essential for Th17 differentiation, and Foxp3, central to Treg identity. IL-6 together with TGF-β induces Th17 differentiation, whereas TGF-β alone promotes Treg differentiation, with HBP functioning as a potential integrator of these cytokine cues [69].

Within the CRC TME, T cell function is suppressed through metabolic competition and signaling disruption. Microbiota-derived metabolites such as deoxycholic acid (DCA) and TCA inhibit CD8^+^ T cell effector activity by impairing Ca^2+^-NFAT signaling [70]. Tumor cells further limit T cell activity by consuming critical nutrients, including glucose and tryptophan [71]. Accumulated lactate from tumor glycolysis acidifies the TME and reinforces immunosuppression. Lactate induces TXNIP expression through the MondoA transcription factor in both CD8^+^ T cells and Tregs. Activation of the MondoA-TXNIP axis diminishes CD8^+^ T cell function by restricting glucose uptake and TCR signaling, while concurrently preserving Treg suppressive capacity [70]. This dual effect illustrates the intricate metabolic interactions shaping immune regulation in the TME.

Moreover, intrinsic metabolic pathways further contribute to shaping T cell activity. S-adenosylmethionine (SAM), the universal methyl donor for histone methylation, sustains the epigenetic framework required for CD8^+^ T cell activation and effector functions, including IFN-γ secretion. Within the TME of MSS CRC, cancer cells deplete extracellular SAM through competitive uptake, leading to diminished histone methylation in T cells and subsequent attenuation of antitumor immunity [72].

Ammonia (NH3/NH_4_^+^), generated as a metabolic byproduct of T cells—primarily through amino acid catabolism such as glutamine and aspartic acid deamination—directly alters T cell metabolic programs and induces exhaustion markers (e.g., PD-1, TIM-3), thereby limiting proliferation and cytokine output. In CRC TME, intensified ammonia metabolism by cancer cells drives ammonia accumulation, a condition clinically linked to poor prognosis and immunotherapy resistance [73].

Furthermore, differentiation and effector activity of T cells, especially inflammatory subsets such as Th17 and cytotoxic T cells, depend on elevated glycolytic flux, whereas memory T cells and Tregs rely predominantly on oxidative phosphorylation. Within the competitive microenvironment of CRC, glucose depletion and lactate accumulation caused by cancer cells and immunosuppressive populations jointly suppress effector T cell activity while favoring Treg-driven immunosuppressive responses [74].

### 2.5. B cells

Within tumors, tertiary lymphoid structures (TLSs) function as specialized niches for B-cell maturation and differentiation, typically appearing as circular or oval aggregates of lymphocytes [75,76]. In the TME, TLSs generally comprise a B-cell zone encircled by a T-cell zone enriched with CD4+ and CD8+ T cells, DCs, and NK cells [77].

In CRC, CD20^+^ B cells and CD138^+^ plasma cells represent the predominant infiltrating B-cell populations [78]. Upon antigen stimulation, CD20^+^ naive or memory B cells recognize antigens via B cell receptors (BCRs) and undergo activation and clonal expansion, driven by costimulatory interactions with CD4^+^ T cells (e.g., CD40L–CD40) and cytokines such as IL-4 and IL-21. This process induces antibody class switching, leading to differentiation into plasma cells capable of producing large quantities of IgG antibodies [79].

B cells, as specialized antigen-presenting cells (APCs), are recruited to the TME via factors such as CXCL13 and CCL21 [80,81]. Antigen recognition occurs either directly through BCR engagement with tumor-related molecules or via activation through pattern recognition receptors such as TLRs, enabling B cells to sense tumor-derived signals [82–84]. Moreover, B cells can trigger tumor cell apoptosis through expression of TNFSF ligands. By internalizing antigens via surface immunoglobulins, even at low concentrations, and processing them with lysosomal enzymes, B cells demonstrate greater efficiency than DCs and TAMs in presenting scarce antigens to T cells [85,86]. The infiltration of B cells, particularly memory B cells, together with TLS formation, shows strong association with enhanced responsiveness and survival benefit in patients treated with PD-1/PD-L1 or anti-CTLA-4 therapies such as Ipilimumab [87,88]. Beyond serving as biomarkers of ICB efficacy, B cells also represent potential therapeutic targets, and modulation of B-cell activity or induction of TLS formation may improve therapeutic outcomes.

Wang et al. [89] identified a previously unrecognized B-cell subset with high leucyl-tRNA synthetase 2 (LARS2) expression, designated LARS B cells, characterized by regulatory activity primarily driven by TGF-β1. These cells share features with plasma cell precursors, including expression of the plasma cell transcriptional regulator Prdm1, yet display limited antibody secretion and reduced CD138 expression. In contrast, they strongly express the inhibitory factor Tgfb1 and maintain extensive interactions with Tregs. LARS2 B cells also exhibit enhanced mitochondrial aminoacyl-tRNA biosynthesis, a metabolic trait potentially linked to reduced survival in CRC patients. Elevated levels of HOTAIR in tumor-derived exosomes have been directly correlated with increased infiltration of PD-L1^+^ B lymphocytes within the TME. Mechanistic studies demonstrate that exosomal HOTAIR interacts with pyruvate kinase M2 (PKM2), inducing STAT3 phosphorylation and subsequent upregulation of PD-L1, thereby endowing B cells with immunosuppressive properties that inhibit cytotoxic CD8+ T-cell activity [90]. This exosome-driven mechanism highlights a novel axis of B-cell-mediated immune evasion and outlines a molecular basis for HOTAIR-targeted therapeutic intervention in cancer.

### 2.6. Natural killer cells

Natural killer (NK) cells, a subset of innate lymphoid cells, exhibit significant heterogeneity within the NK cells, belonging to the innate lymphoid lineage, display considerable heterogeneity within the TME and possess intrinsic cytolytic capacity against tumor cells, thereby inhibiting proliferation, migration, and colonization [91]. Based on differential expression of surface markers, NK cells are stratified into CD56^bright^CD16^low/-^ and CD56dimCD16+ subsets. The former primarily fulfills immunoregulatory roles through cytokine secretion, whereas the latter demonstrates potent cytotoxic potential [92].

Recognition of antibody-coated tumor cells through Fc receptors initiates targeted degranulation and direct lysis of tumor cells. Loss of N1-type TANs diminishes NK cell activity, reducing responsiveness to antibody-coated targets and weakening cytotoxic efficacy [26]. NK cell–derived cytokines such as IFN-γ and TNF-α enhance the antitumor capacity of TAMs and T cells, thereby reinforcing coordinated immune responses. Experimental evidence indicates that IL-2 enhances the cytotoxic effect of NK-92 cells against SW480 cells, increases IFN-γ secretion, and modulates CRC cell growth and apoptosis via regulation of IL-15 [93]. In addition, NK cells induce apoptosis through the release of perforin- and granzyme-containing granules [27]. Tumor cells, however, can escape NK-mediated surveillance by reducing or eliminating ligands of activating receptors, including MHC class I polypeptide–related molecules [94].

Emerging evidence indicates that mesenteric lymph node NK cells in CRC display elevated expression of T-cell immunoglobulin and mucin domain-3, lymphocyte activation protein-3, PD-1, and T-cell immunoreceptor with immunoglobulin or ITIM domain, accompanied by reduced levels of TNF-related apoptosis-inducing ligand, along with a marked increase in sirtuin 2 expression. *In vitro* analyses have further shown that sirtuin 2 attenuates the tumor-suppressive capacity of exhausted NK cells [95]. Moreover, downregulation of zinc finger protein 335 has been demonstrated to significantly impair NK cell cytotoxicity in murine models of colitis-associated CRC. Silencing of zinc finger protein 335 disrupts NK cell proliferation and decreases the expression of Janus kinase 1 and Janus kinase 3, leading to impaired NK cell homeostasis and diminished functional activity [63]. Collectively, these results highlight novel mechanistic insights and potential therapeutic targets for advancing immunotherapy strategies in CRC.

### 2.7. Cancer-associated fibroblasts

CAFs arise from multiple cellular origins, including tissue-resident fibroblasts, mesenchymal stem cells, and epithelial cells [96]. Activation of CAFs occurs through diverse signaling pathways: TGF-β engages the SMAD cascade and drives the conversion of normal fibroblasts into CAFs via epithelial–mesenchymal transition (EMT) and endothelial–mesenchymal transition [97,98], while IL-1β and IL-6 activate CAFs through NF-κB and JAK-STAT3 signaling. Once activated, CAFs remodel the extracellular matrix (ECM) by synthesizing matrix components and expressing proteases such as fibroblast activation protein (FAP), thereby shaping an environment conducive to TAM infiltration within the TME [99–101]. In addition, CAF-derived granulocyte–macrophage colony-stimulating factor (GM-CSF) and IL-8 actively attract TAMs and drive their polarization toward the M2 phenotype. M2 TAMs, in turn, reinforce CAF activation and induce mesenchymal–mesenchymal transition (MMT), establishing a reciprocal circuit that maintains tumor-promoting conditions and enhances CAF responsiveness [102,103].

Evidence indicates that fibroblast activation is limited during early tumorigenesis but becomes prominent in later stages, when malignant cells acquire invasive and metastatic traits. The involvement of CAFs in CRC therefore marks progression toward advanced or terminal malignant transformation. Once activated, CAFs are regulated by TGF-β receptor signaling, which engages alternative pathways through p38-MAPK-mediated mechanisms. This signaling cascade drives fibroblast secretion of mitogenic factors and regulatory molecules, thereby accelerating tumor cell transition from G1 to S phase and enhancing proliferative capacity [104].

Koncina et al. [105], using single-cell sequencing, identified distinct CAF subpopulations with immunosuppressive activity, highlighting the role of IL-1R1+ CAFs in promoting tumor expansion. Increased IL1R1 expression correlated with altered immune cell signatures and elevated exhaustion markers such as FAP and CXCL12. High expression levels observed in CMS4 CRC tissues were linked to unfavorable outcomes, suggesting that therapeutic strategies targeting stromal components of the TME may provide clinical benefit and improve prognosis in CRC.

### 2.8. Extracellular matrix

The ECM constitutes a three-dimensional scaffold of proteins and polysaccharides surrounding cells, providing structural stability, regulating differentiation, and serving as a reservoir for growth factors that modulate proliferation and migration [106–109].

In CRC, ECM composition undergoes extensive remodeling compared with normal colon tissue. Laminin and type IV collagen expression is commonly diminished, particularly in metastatic lesions where basement membrane integrity is disrupted, whereas type I collagen deposition increases, correlating with heightened tissue stiffness and unfavorable prognosis. Concurrently, hyaluronic acid, fibronectin, elastin, and VEGF are frequently enriched. Accumulated hyaluronic acid plays a central role in shaping an immunosuppressive milieu by recruiting M2 macrophages and limiting T-cell infiltration. These alterations reshape mechanical properties, reprogram cellular signaling, and reinforce metastatic potential, collectively driving tumor advancement [110–112].

Beyond encoding the SERPINE1 protein, Serpine1 mRNA acts as a regulatory noncoding RNA that accelerates EMT, enhances migratory and invasive behavior, and supports cell survival. Elevated Serpine1 expression in CRC correlates with enhanced tumor progression and dissemination, while clinical analyses demonstrate an inverse relationship between SERPINE1 mRNA levels and CD8^+^ T-cell infiltration, highlighting its potential as a therapeutic target [113].

### 2.9. Others

Extracellular vesicles (EVs) act as essential mediators of intercellular communication between tumor and host cells, carrying diverse biomolecules and RNA species. By modulating cellular activity and gene expression, EVs influence immune dynamics within CRC, particularly the polarization of TAMs. CRC-derived sEVs transfer miR-21-5p and miR-200a into TAMs, suppressing PTEN/AKT and SOCS1/STAT1 signaling, which drives macrophages toward an immunosuppressive M2 phenotype characterized by elevated PD-L1 expression, thereby promoting immune evasion and tumor progression [114]. Uptake of EVs by monocytes and macrophages further enhances CD14 expression in M0 macrophages while reducing HLA-DR expression in both M1 and M2 subsets, fostering a proinflammatory and tumor-supportive TAM phenotype [115].

Exosomes, membrane-bound vesicles approximately 30–100 nm in size, are secreted by many cell types, including mast cells and DCs [116]. Tumor cells release larger quantities of exosomes than normal cells, and in CRC, exosomal miR-1246 has emerged as a key regulator of TAM reprogramming, reinforcing immune tolerance and enhancing invasive and migratory potential [39,117]. Derived from the RNU2-1 fragment, exosomal miR-1246 induces macrophage polarization toward M2-like TAMs. Its packaging into exosomes is mediated by hnRNPA2B1, which recognizes the Exo motif within miR-1246, while TUT7 uridyltransferase augments exosomal miR-1246 levels by catalyzing uridylation of small noncoding RNAs, collectively shaping TAM polarization and accelerating CRC progression [39].

**Figure 1.**
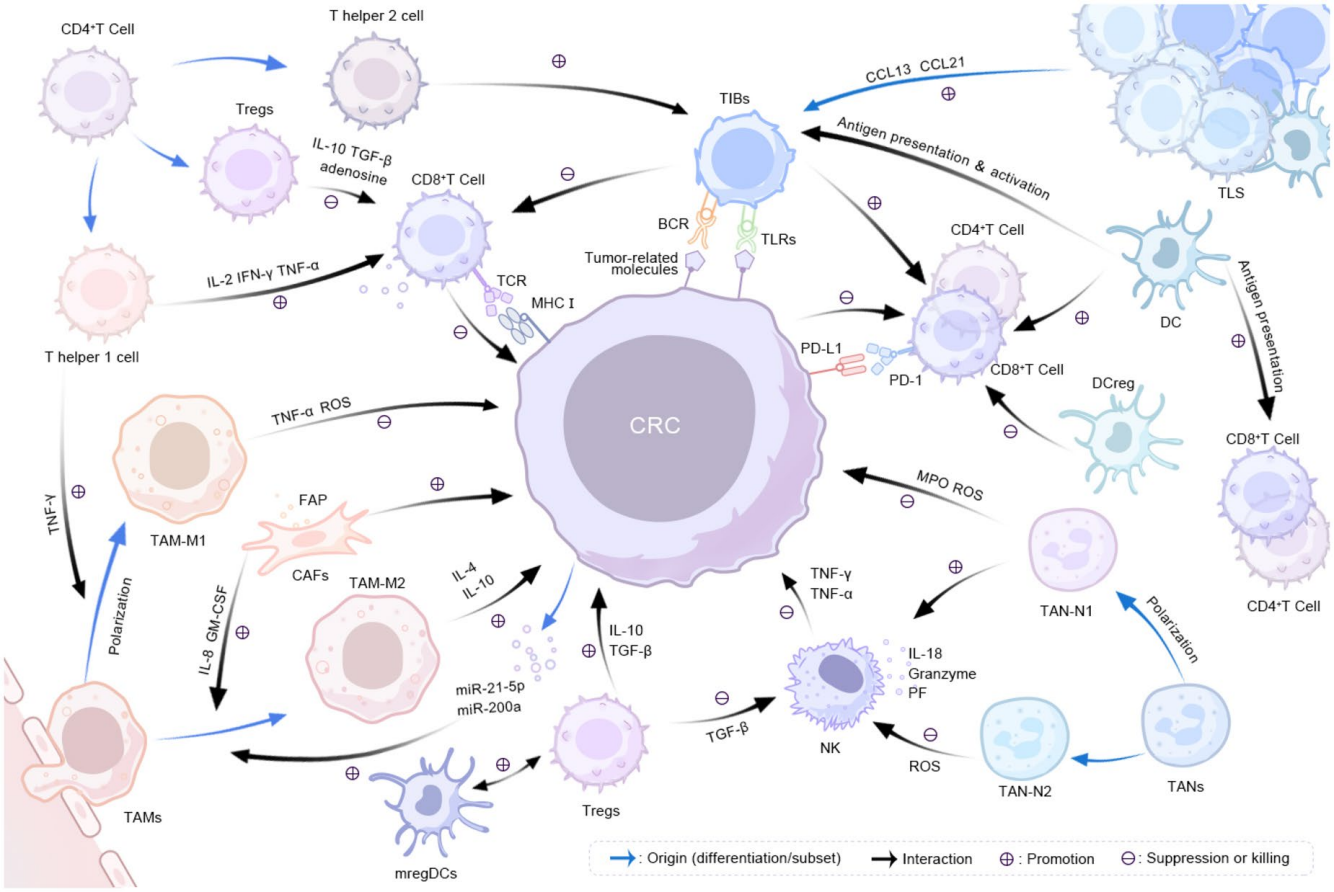
Cellular interactions in the colorectal cancer immune microenvironment. Blue arrows denote sources, such as differentiation or subsets. Black arrows indicate interactions between these two cell types, with a ‘+’ signifying promotion and a ‘–’ signifying inhibition or killing.

Overall, the CRC immune microenvironment includes diverse cellular components, including TAMs, TANs, CAFs, T cells, and NK cells, forming an intricate network that governs tumor immunity. A detailed mechanistic summary is presented in Figure 1.

## 3. Cutting-edge clinical research on immunotherapy for CRC

### 3.1. Studies on immune checkpoint therapy in CRC

Immunotherapy has become the fourth principal therapeutic approach for cancer, following surgery, chemotherapy, radiotherapy, and targeted therapy. Its central strategy relies on immune checkpoint inhibition, where immune checkpoint inhibitors (ICIs) restore antitumor responses by blocking inhibitory receptors on immune cells. By targeting receptors such as PD-1(in CD8^+^ T cell or CD4^+^ T cell) and CTLA-4 (in Naive CD4^+^ T cell) or their ligands (e.g., PD-L1), ICIs mitigate immunosuppressive signaling within the tumor microenvironment, thereby reactivating T cell activation and proliferation, enabling renewed recognition and elimination of tumor cells, and ultimately exerting antitumor effects [118–120].

ICIs directed against PD-1/PD-L1 and CTLA-4 have been applied in CRC therapy, yielding marked efficacy in patients with DNA mismatch repair (dMMR) deficiency or microsatellite instability-high (MSI-H) tumors. Combination therapy with nivolumab and ipilimumab has proven effective in cases of rapidly progressing CRC. Despite the clinical benefit observed with both monotherapy and dual-agent ICIs in MSI-H and dMMR tumors, responses to single-agent ICIs in microsatellite stable (MSS) and mismatch repair-proficient (pMMR) tumors remain comparatively limited [121–123].

The therapeutic effect of ICIs relies on immune recognition of tumor-derived neoantigens, which activates effector T cells to eliminate malignant cells [124]. However, MSS/pMMR CRC exhibits a non-inflammatory TME characterized by low tumor mutation burden (TMB) and deficient antigen presentation [125]. Even when limited neoantigens are present, immune responses remain suppressed within MSS/pMMR CRC through mechanisms such as inadequate infiltration and functional exhaustion of effector T cells, enrichment of immunosuppressive cell populations, and accumulation of inhibitory cytokines and metabolites, thereby limiting the efficacy of ICIs [126].

To address resistance in MSS/pMMR CRC, diverse combinatorial approaches are under investigation. Current clinical studies primarily evaluate ICI-based regimens incorporating anti-angiogenic agents, chemotherapy, or dual checkpoint inhibitors, aiming to remodel the TME and increase neoantigen release. In advanced MSS/pMMR CRC, treatment with sintilimab plus bevacizumab and chemotherapy increased CD8+ T-cell infiltration while reducing TAMs and CAFs [127]. Likewise, biomarker analyses from the REGONIVO and TASNIVO phase Ib trials demonstrated that regorafenib combined with nivolumab achieved a median PFS of 7.9 months in a subset of MSS mCRC patients, an effect associated with depletion of Tregs and M2 macrophages [128]. In a phase II study, cetuximab plus avelumab produced clinical benefit in MSS/pMMR RAS WT metastatic CRC, yielding a median OS of 11.6 months and a disease control rate of 64.8% with manageable toxicity. Notably, in 44 MSS patients with baseline RAS/BRAF WT ctDNA, median OS extended to 17.3 months, supporting the refinement of ICI-based strategies through rational drug combinations and ctDNA-guided patient selection [129].

Beyond the PD-1/CTLA-4 axis, efforts are directed toward characterizing additional immune checkpoints such as LAG-3 and optimizing CTLA-4 blockade to improve therapeutic efficacy in CRC, especially in CD8^+^ T cell. CTLA-4, a key inhibitory receptor on T cells, attenuates early immune activation by binding CD80/86 with higher affinity than CD28. In microsatellite-stable (MSS) rectal cancer, which shows limited responsiveness to ICIs, the phase II CHINOREC trial demonstrated that incorporating neoadjuvant ipilimumab (anti-CTLA-4) and nivolumab (anti-PD-1) into chemoradiotherapy (CRT) was safe and feasible, without elevating surgical risks; however, no significant improvement in pathological complete response (pCR) was observed compared with CRT alone (22% vs 30%) [130]. A phase Ib trial is currently evaluating neoadjuvant sintilimab (anti-PD-1) combined with hypofractionated radiotherapy (25 Gy/5 fractions) in locally advanced MSI-H/dMMR rectal cancer, reporting a 60% pCR rate and manageable toxicity, suggesting considerable potential in this subgroup [131].

LAG-3, an inhibitory receptor expressed on lymphocytes(such as activated effector T cells, especially ‘exhausted’ CD8^+^T cells), suppresses effector activity through interaction with MHC class II molecules. The NICHE-3 phase II trial assessed neoadjuvant nivolumab (anti-PD-1) with relatlimab (anti-LAG-3) in locally advanced dMMR CRC, achieving pathological responses (RVT ≤ 50%) in 97% of patients, including a 68% pCR rate, while maintaining acceptable safety. Mechanistic investigations revealed that ligand-induced ubiquitination activated the LAG-3 checkpoint function, and LAG-3/CBL co-expression has emerged as a potential biomarker for predicting therapeutic responsiveness to LAG-3 inhibition [132].

Immune checkpoint blockades (ICBs) primarily function by restoring or enhancing antitumor immunity through inhibition of checkpoint interactions between tumor and immune cells. While ICBs promote T-cell activation by blocking checkpoint signaling, ICIs achieve similar effects via checkpoint inhibition. Recent investigations have focused on strategies to overcome immune evasion in ‘cold’ tumors, stimulate immune activity within the tumor microenvironment, and improve tumor sensitivity to immunotherapy, particularly in combination regimens. Substantial clinical benefit has been documented in MSI-H CRC, where ICB therapy is strongly recommended for patients with dMMR/MSI-H mCRC. In contrast, pMMR/MSS tumors generally remain unresponsive due to intrinsic resistance to immunotherapy, and ICB administration is not advised in this setting [133,134].

Pembrolizumab has been established as the first-line standard treatment for MSI-H/dMMR metastatic CRC, with superiority over conventional chemotherapy. In addition, nivolumab combined with ipilimumab as initial therapy in metastatic CRC with MSI-H/dMMR significantly extended progression-free survival (PFS) compared with chemotherapy. Long-term follow-up from the KEYNOTE-177 trial confirmed that first-line pembrolizumab markedly improved both overall survival (OS) and PFS relative to chemotherapy while also lowering the incidence of high-grade adverse events [135–137].

Recent investigations indicate that combining ICIs with other therapeutic modalities can improve treatment efficacy, with ICI–chemotherapy regimens demonstrating particularly promising results in several cancer types. In MSS metastatic CRC (mCRC), integration of nivolumab with the FOLFOX regimen has been associated with extended progression-free survival and higher objective response rates compared with chemotherapy alone, suggesting that short-course oxaliplatin-based chemotherapy may sensitize MSS mCRC patients to ICIs [138]. An open-label phase 1b/2 trial assessed the combination of the mek1/mek2 inhibitor binimetinib with nivolumab or with nivolumab plus ipilimumab in RAS-mutant MSS metastatic CRC. Although toxicity profiles were acceptable, the clinical benefit was limited, and overall response rates remained lower than those achieved with nivolumab alone or in combination with ipilimumab [139].

Preclinical evidence from a randomized phase 2 study further examined sintilimab combined with the histone deacetylase inhibitor chidamide, with or without the anti-VEGF antibody bevacizumab, in patients with unresectable, chemotherapy-refractory locally advanced CRC or pMMR/MSS mCRC. Results suggested that combined chemotherapy and immunotherapy could attenuate primary resistance to ICI monotherapy in pMMR/MSS mCRC. Triple therapy with sintilimab, chidamide, and bevacizumab demonstrated notable therapeutic activity in CRC patients irrespective of liver metastasis status [140].

Immunogenic chemotherapy has been shown to enhance the efficacy of ICIs by promoting tumor infiltration of CD4^+^ and CD8^+^ T cells while attenuating the activity of immunosuppressive populations such as Tregs. MS4A4A represents a promising target in this context, as blockade of MS4A4A activates PI3K/AKT and JAK/STAT6 signaling, driving TAM polarization toward the M2 phenotype, decreasing infiltration of exhausted T cells, and enhancing effector CD8^+^ T-cell infiltration, thereby enhancing the therapeutic effects of ICIs. Combined inhibition of MS4A4A and PD-1 has demonstrated substantial antitumor efficacy in drug-resistant CRC, significantly delaying tumor progression and indicating its potential as a novel immunotherapeutic approach [141].

Approximately 25% of MSI-H CRC patients display intrinsic resistance to immunotherapy, with objective remission rates (ORR) of 38% for anti-PD-1 monotherapy and 54% for anti-PD-L1 monotherapy, while anti-PD-1 combined with anti-CTLA-4 therapy achieves the highest ORR of 57% [142]. LAG-3 has recently emerged as a target of considerable interest. Evidence suggests that co-blockade of PD-1 and LAG-3 holds therapeutic potential in CRC. In MSS MCRC, evaluation of the anti-LAG-3 antibody BI754111 in combination with the anti-PD-1 antibody BI754091 demonstrated disease stabilization in a subset of patients, highlighting the feasibility of this strategy. Although immunotherapy for CRC remains in an exploratory phase, the integration of PD-1 and LAG-3 inhibition offers a promising avenue for improving clinical outcomes [143].

**Figure 2.**
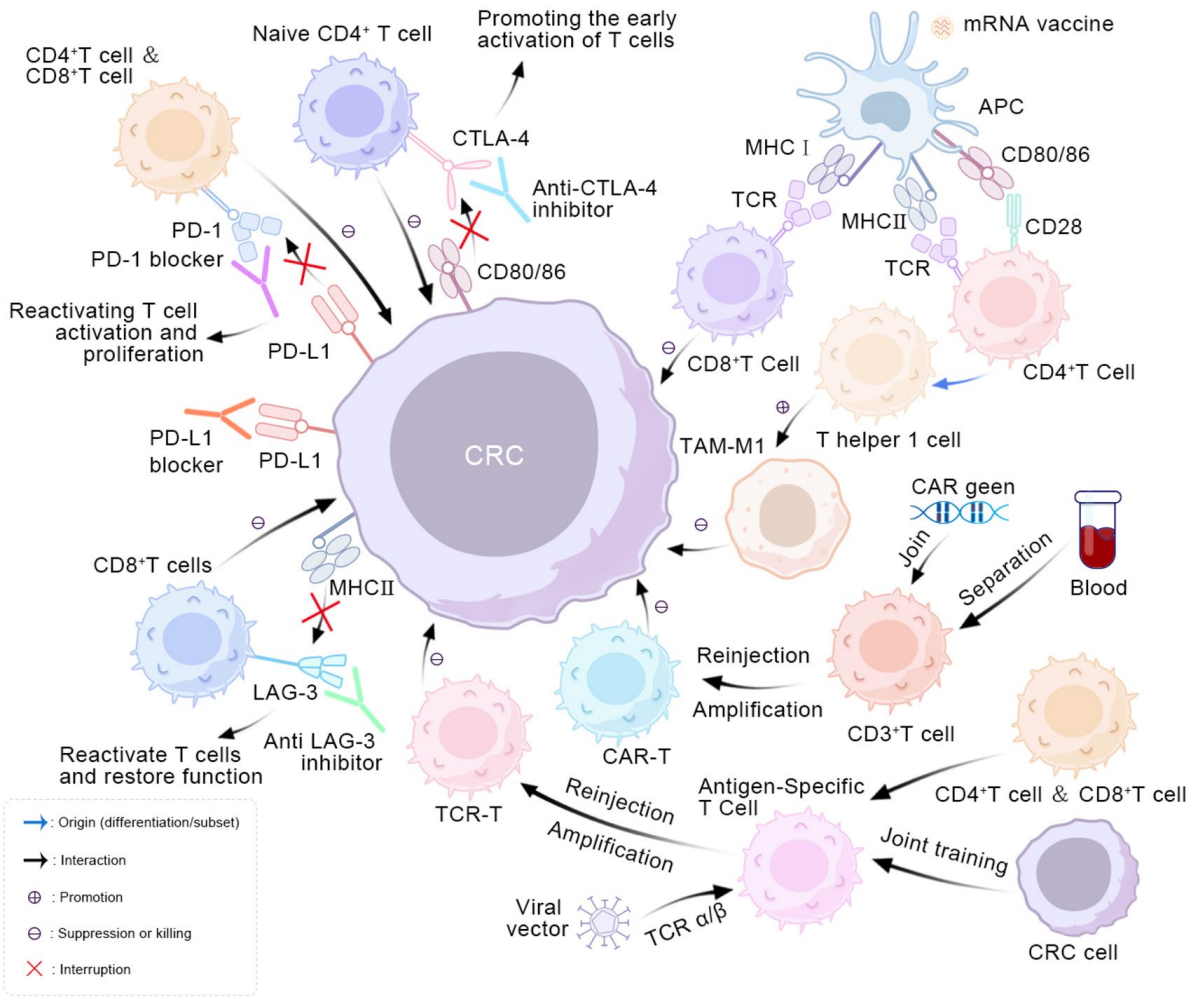
Mechanistic diagram of immunotherapy for colorectal cancer. Blue arrows denote sources, such as differentiation or subsets. Black arrows indicate interactions between these two cell types, with a ‘+’ signifying promotion and a ‘–’ signifying inhibition or killing. The red cross indicates that this process has been interrupted.

Advances in biological and immunological research continue to expand the landscape of immune checkpoint-based therapies, and the integration of checkpoint blockade with additional therapeutic modalities is expected to define an important trajectory for future research (Figure 2; Table 1).

**Table 1.** Types, processes, results and conclusions of ICIs clinical trials

Target/combination	Clinical phase	Treatment	Results	Conclusion	References
Sintilimab and bevacizumab	Phase II clinical trial	110 MSS/pMMR advanced CRC patients after 1st-line failure; 60 in experimental group (Sintilimab + Bevacizumab), 50 in control group (FOLFIRI + Bevacizumab); 4 cycles. Evaluated CD8^+^ T cells, TAMs, CAFs, efficacy, and safety.	a. CD8^+^ T cells: positive rate in experimental group (50% vs 30% control). b. TAMs/CAFs: positive rates in experimental group (TAMs: 23.3% vs 60%,; CAFs: 23.3% vs 50%). c. Efficacy: Higher ORR in experimental group (26.7% vs 10%). No significant difference in DCR or PFS. d. Safety: No significant difference in adverse events between groups.The combination of Sintilimab (ICI) and Bevacizumab (anti-angiogenic) improved the tumor immune microenvironment (increased CD8^+^ T cells, decreased TAMs/CAFs) and clinical efficacy (ORR) in MSS/pMMR advanced CRC patients after 1st-line failure, with a manageable safety profile comparable to chemotherapy.	The combination of Sintilimab (ICI) and Bevacizumab (anti-angiogenic) improved the tumor immune microenvironment (increased CD8^+^ T cells, decreased TAMs/CAFs) and clinical efficacy (ORR) in MSS/pMMR advanced CRC patients after 1st-line failure, with a manageable safety profile comparable to chemotherapy.	[127]
Cetuximab and avelumab	Phase II clinical trial	Enrolled 77 RAS WT mCRC patients (71 MSS, 3 MSI-H, 3 unknown) who responded to first-line anti-EGFR therapy, progressed, and failed second-line therapy; treated with cetuximab (400 mg/m² loading, then 250 mg/m² weekly) + avelumab (10 mg/kg q2w) until progression/toxicity; assessed efficacy via OS/PFS/ORR, safety via NCI-CTC v4.03, and ctDNA (KRAS/NRAS/BRAF/EGFR-S492R) via Idylla qPCR	a. ITT population: mOS = 11.6 months, mPFS = 3.6 months, DCR = 65%, ORR = 7.8% b. 71 MSS patients: mOS = 11.6 months, mPFS = 3.6 months, DCR = 64.8% c. 44 MSS patients with baseline RAS/BRAF WT ctDNA: mOS = 17.3 months, mPFS = 3.9 months, DCR = 72.7% d. Common grade 3 AEs: cutaneous eruption (14%), diarrhea (4%); no grade 4/5 AEs	Cetuximab + avelumab is active and well-tolerated as rechallenge for RAS WT mCRC(92%MSS); baseline plasma RAS/BRAF WT ctDNA selects patients likely to benefit	[129]
KEYNOTE-177	Phase III clinical trial	a. The trial was conducted across 193 academic medical centers and hospitals in 23 countries globally. b. Patients were randomly assigned (1:1) to receive either pembrolizumab (200 mg administered intravenously every three weeks) or chemotherapy (using modified FOLFOX6 or FOLFIRI, with or without bevacizumab or cetuximab).	a. The incidence of adverse events (AEs) was lower in the pembrolizumab group than in the chemotherapy group, particularly for grade 3–5 AEs (22% vs 67%). b. No treatment-related deaths occurred in the pembrolizumab group, in contrast to one death attributed to bowel perforation in the chemotherapy group. c. In the pembrolizumab group, the most frequent treatment-related adverse events were diarrhea and fatigue, whereas in the chemotherapy group, they included nausea, diarrhea, and fatigue.	Over 50% of patients in the pembrolizumab group demonstrated long-term survival with follow-up exceeding 5 years. The sustained efficacy and safety profile of pembrolizumab supports its adoption as a standard first-line therapy for patients with MSI-H/dMMR mCRC.	[135]
Binimetinib, nivolumab, ipilimumab	Phase Ib/2 clinical trial	a. Phase 1b: Participants were randomized to either Arm 1A (binimetinib 45 mg twice daily [BID] plus nivolumab 480 mg every 4 weeks [Q4W]) or Arm 1B (binimetinib 45 mg BID plus nivolumab 480 mg Q4W and ipilimumab 1 mg/kg every 8 weeks [Q8W]) to establish the maximum tolerated dose (MTD) and recommended phase 2 dose (RP2D). b. Phase 2: Participants were randomly assigned to Arm 2A (binimetinib at the MTD/RP2D plus nivolumab) or Arm 2B (binimetinib at the MTD/RP2D plus nivolumab and ipilimumab) for the evaluation of the safety and clinical activity of these combinations.	a. Seventy-four of 75 participants (98.7%) experienced treatment-related adverse events (AEs), including 17 (22.7%) with serious AEs. The most common treatment-related AEs were dermatitis acneiformis, elevated blood creatine phosphokinase, diarrhea, fatigue, and peripheral edema. b. Regarding efficacy, in Phase 1b, no objective responses were observed in either Arm 1A or Arm 1B. In Phase 2, none of the participants receiving binimetinib plus nivolumab achieved a response, whereas among the 27 participants treated with binimetinib, nivolumab, and ipilimumab, the objective response rate (ORR) was 7.4% (90% CI: 1.3–21.5).	The combination of binimetinib with nivolumab or nivolumab plus ipilimumab demonstrated a safety profile consistent with previous studies in patients with MSS-type metastatic colorectal cancer (mCRC) harboring RAS mutations, but did not demonstrate significant clinical benefit.	[139]
Sintilimab,chidamide,bevacizumab	Phase II clinical trials	a. A total of 48 patients were enrolled and randomly allocated in a 1:1 ratio to either the dual therapy group (*n* = 23) or the triple therapy group (*n* = 25). b. The dual therapy group received sintilimab combined with chidamide, while the triple therapy group received additional bevacizumab. Significantly superior efficacy was observed in the triple therapy group compared to the dual therapy group, with 18-week PFS rates of 64.0% versus 21.7% (*P* = 0.003), median PFS of 7.3 months versus 1.5 months (*P* = 0.006), and ORR of 44.0% versus 13.0% (*P* = 0.027), respectively.	The triple-therapy group demonstrated significantly superior outcomes compared to the double-therapy group, with 18-week progression-free survival (PFS) rates of 64.0% versus 21.7% (*P* = 0.003), median PFS of 7.3 months versus 1.5 months (*P* = 0.006), and objective response rates (ORR) of 44.0% versus 13.0% (*P* = 0.027), respectively.	The triple combination therapy, consisting of the PD-1 antibody sintilimab, the HDAC inhibitor chidamide, and the anti-VEGF antibody bevacizumab, demonstrated significant efficacy in patients with colorectal cancer of the MSS/pMMR phenotype. Notably, it substantially outperformed the double combination therapy across multiple endpoints, including progression-free survival, objective remission rate, and disease control rate.	[140]
BI754111 and BI754091	Phase I clinical trials	MSS mCRC patients were treated with combination therapy consisting of the anti-LAG-3 antibody BI754111 and the anti-PD-1 antibody BI754091.	Among the enrolled patients, a subset achieved stable disease (SD); however, the overall response rate (ORR), defined as the combination of complete and partial responses (CR+PR), remained limited.	This study offers valuable foundational data and insights for the further investigation of more effective immunotherapy strategies for colorectal cancer, particularly in addressing the challenge of immunotherapy resistance.	[143]

### 3.2. Research on tumor vaccine therapy

Tumor vaccines provide a less toxic alternative to chemotherapy and radiotherapy and include diverse forms, including cell-based vaccines (e.g., DC [144] or T-cell [145] vaccines), viral vaccines [146], bacterial vaccines [147], and molecular platforms such as DNA [148] and mRNA [149] vaccines. Their therapeutic effect relies on initiating antigen-specific immune responses against tumor-associated antigens (TAAs) or tumor-specific antigens (TSAs) expressed by cancer cells. By promoting antigen presentation, these vaccines activate immune cells to identify and eliminate malignant cells [150]. TAAs, defined as nonmutated self-proteins aberrantly expressed in tumors, include overexpressed molecules (e.g., HER2, MUC-1), differentiation antigens (e.g., tyrosinase), and carcinoembryonic antigens such as the MAGE family [151]. TSAs, in contrast, are expressed exclusively in tumor cells and comprise mutation-derived neoantigens as well as viral oncogene products, including HPV E6/E7. Owing to their high tumor specificity and strong immunogenic potential, TSAs represent key targets for cancer vaccine development [152].

The mRNA vaccines employ carriers such as lipid nanoparticles (LNPs) to encapsulate mRNA, protect it from enzymatic degradation, and enhance cellular internalization. Following endocytic uptake, mRNA is released into the cytoplasm, where it undergoes direct translation into antigenic proteins [153,154]. These proteins, together with tumor vaccines, introduce TAAs and mutation-derived neoantigens that are captured and processed by APCs, particularly dendritic cells. The processed antigens are presented on MHC molecules to T cells: MHC class I presents to CD8^+^T cells, and MHC class II presents to CD4^+^T cells. This presentation, specifically the engagement of the TCR by peptide-MHC complexes, provides the primary activation signal (signal 1). A second, costimulatory signal (signal 2) is concurrently provided by the interaction between receptors on T cells (such as CD28) and their ligands on APCs (such as B7(CD80/86)molecules), leading to robust T cell activation [152,155]. Activated CD8^+^ T cells recognize antigen–MHC complexes expressed on tumor cells and execute direct cytotoxicity, whereas CD4^+^ T cells differentiate predominantly into T helper type 1 (Th1) cells, which promote CD8^+^ T cell-mediated tumor elimination and reinforce immune memory through cytokine secretion. These helper T cells, along with cytotoxic T cells, further amplify the immune cascade by releasing cytokines such as IL-2 and IFN-γ. Combination approaches, such as adjuvants and ICIs, counteract tumor-induced immunosuppression, intensify inflammatory signaling, and recruit additional immune subsets for coordinated antitumor activity. Moreover, vaccine-induced memory T cells establish durable immune surveillance, while antigen spreading extends the breadth of tumor recognition and destruction [156–158].

Substantial progress has been achieved in CRC vaccine research, with emphasis on KRAS mutation-directed vaccines, MSS-type peptide vaccines, and personalized neoantigen vaccines, where integration with immune checkpoint blockade or chemotherapy markedly enhances therapeutic efficacy.

ELI-002 2P is a lymph node-targeting cancer vaccine composed of amphiphilicity (Amph)-modified KRAS^G12D^ and KRAS^G12R^ mutant peptides (Amph-peptides-2P) combined with the CpG oligonucleotide adjuvant Amph-CpG-7909. This formulation enhances lymph node delivery, induces T-cell recognition of KRAS mutations, promotes clearance of KRAS-mutated cancer cells, and lowers the risk of tumor recurrence. A clinical trial in CRC patients reported biomarker clearance in a subset of participants treated with a fixed dose of Amph-peptide-2P and escalating doses of Amph-CpG-7909, indicating therapeutic potential [159].

Another immunotherapeutic approach involves codelivery of all-trans retinoic acid (ATRA) with an mRNA vaccine via LNPs. This strategy markedly increased mRNA transfection efficiency *ex vivo* and induced T-cell expression of intestinal homing receptors CCR9 and α4β7, thereby strengthening mucosal immune responses. In an in situ CRC mouse model, the ATRA-assisted mRNA vaccine significantly suppressed tumor growth and extended survival without notable toxicity, supporting its promise as a preclinical immunotherapy candidate [160].

Personalized neoantigen-directed therapy GRANITE, which integrates a chimeric adenovirus with a self-amplifying mRNA vaccine, demonstrated superior PFS over standard first-line treatment in patients with newly diagnosed MSS-CRC, particularly those with low tumor burden, in a randomized phase II clinical trial. Neoantigen-specific T-cell responses were consistently observed across evaluable patients, and treatment-related adverse events remained manageable. These results suggest potential benefit of GRANITE in first-line MSS-CRC therapy, especially for individuals with limited disease burden [161].

A recent study on CRC immunotherapy developed an αDC1 vaccine targeting the tumor pericyte antigen DLK1. In a homozygous mouse model, this vaccine enhanced cytotoxic T lymphocyte activity, disrupted tumor vasculature, and effectively suppressed colon cancer progression, thereby establishing an experimental foundation for DC1-based immunotherapy in CRC [162].

Despite obstacles posed by tumor heterogeneity and immunosuppressive microenvironments, personalized vaccines, such as neoantigen-based mRNA platforms, are anticipated to represent a major direction in precision oncology. Advances in antigen selection, delivery technologies, multitargeting approaches, and combination regimens are expected to strengthen their therapeutic potential. Ongoing clinical progress in CRC vaccine development is outlined below (Figure 2; Table 2).

**Table 2.** Stages, treatments, results, and conclusions of clinical/preclinical trials of colorectal cancer tumor vaccines

Name	Stages	Treatments	Result	Conclusion	References
ELI-002 2P	Phase I clinical trials	a. Mechanism of action: The enhancement of lymph node delivery and immune response is mediated by the binding of amphiphile-modified G12D and G12R mutant KRAS (mKRAS) peptides to CpG oligonucleotide adjuvant (Amph-CpG-7909) b. The regimen comprised fixed-dose Amph-Peptides-2P (G12D and G12R peptides 0.7 mg each) administered in combination with increasing doses of Amph-CpG-7909 (0.1 mg to 10.0 mg)	a. Direct *in vitro* mKRAS-specific T-cell responses were detected in 84% of patients (21/25) b. Tumour biomarker decline was observed in 84% of patients (21/25), with biomarker clearance achieved in 24% of patients (6/25)	The study included 25 patients (20 with pancreatic cancer and 5 with colorectal cancer). The small cohort of colorectal cancer patients underscores the need for further research to evaluate the potential efficacy of this treatment for tumours.	[159]
ATRA-assisted mRNA vaccines	Pre-clinical studies	a. Co-delivery of ATRA and mRNA vaccines encoding tumour antigens was performed using LNP as the delivery vehicle. b. The vaccine was administered to mice via intramuscular injection. mRNA expression was monitored using the *in vivo* imaging system for small animals (IVIS), and immune cell activation along with tumour infiltration were analysed by flow cytometry. c. An in situ colorectal cancer tumour model was established to evaluate the inhibitory effect of the vaccine on tumour growth and mouse survival.	a. ATRA enhanced mRNA transfection efficiency: ATRA-LNP significantly increased mRNA transfection efficiency *in vitro* and enhanced mRNA expression *in vivo* compared to LNP without ATRA. b. Enhancement of intestinal mucosal immune response: ATRA induced the expression of intestinal homing receptors CCR9 and α4β7 on T cells, and increased the infiltration of antigen-specific T cells into the intestinal lamina propria and mesenteric lymph nodes. c. Inhibition of tumour growth: In an in situ colorectal cancer model, ATRA-LNP significantly inhibited tumour growth and prolonged the survival of mice, whereas conventional LNP vaccines exhibited limited efficacy.	It has potential applications in the treatment of colorectal cancer, but further clinical trials are required to validate the efficacy.	[160]
GRANITE	Randomised phase II clinical study	a. The study enrolled newly diagnosed MSS-CRC patients whose tumors harbored at least 20 neoantigens as screened by the EDGE prediction model. b. Patients were randomly assigned 1:1 to receive either GRANITE therapy (comprising the GRANITE vaccine plus first-line standard of care, i.e., SOC) or first-line standard of care alone in the control group. c. The therapeutic regimen utilized chimeric adenovirus and self-amplifying mRNA vaccines encoding patient-specific neoantigens in conjunction with immune checkpoint inhibitors.	a. Patients in the GRANITE group demonstrated improved PFS relative to controls (HR = 0.73, 90% CI [0.44,1.21]). b. Greater benefit from GRANITE therapy was observed in patients with low disease burden, indicated by a higher proportion of patients being free of disease progression and exhibiting very low levels of circulating tumour DNA (ctDNA). c. Neoantigen-specific T-cell responses were detected in all evaluable patients in the GRANITE group. d. Molecular responses, defined by ctDNA reduction at a single time point, were similar between the two groups (39% in the GRANITE group and 40% in the control group).	This study suggests potential clinical benefit for patients with MSS-CRC, especially those with low disease burden, in the first-line setting.	^[163]^
α DC 1 vaccines	Pre-clinical studies	Tumour models were established by subcutaneous injection of the MC38 colon adenocarcinoma cell line in a homozygous mouse model. Mice were treated with a DC1–DLK1 vaccine in combination with anti-PD-1 antibody to enhance efficacy. Vaccine-induced immune responses and anti-tumour effects were assessed by flow cytometry, immunohistochemistry, immunofluorescence, and quantitative PCR.	a. The DC1 vaccine significantly reduced tumour volume and prolonged survival in mice without significant side effects. b. Vaccine treatment increased the infiltration of cytotoxic T lymphocytes within the tumour, promoted tumour vascular disruption, and decreased tumour vessel density. c. All mice treated with a DC1-DLK1 vaccine exhibited significant anti-tumour effects, and these effects were superior to those of mice treated with DC1 vaccine in combination with anti-PD-1 antibody alone or pulsed with irrelevant peptides.	The DC1 vaccine against the tumour pericyte antigen DLK1 demonstrated significant anti-tumour effects in a homozygous mouse model, providing a new strategy for immunotherapy of CRC.	[162]

### 3.3. Adoptive cell transfer

Adoptive cell transfer (ACT), an advanced modality in cancer immunotherapy, involves *ex vivo* expansion and genetic modification of patient-derived immune cells to enhance their cytotoxicity, followed by reinfusion to eradicate malignant cells. Multiple ACT strategies are under investigation for CRC, including CAR-T therapy, tumor-infiltrating lymphocytes, TCR-engineered T cells, and NK-cell therapy [163]. Both CAR-T and TCR-T therapies involve genetic engineering of patient-derived T cells to potentiate antitumor immunity. It is noteworthy that the initial T cell populations used for engineering differ: CAR-T therapies typically use CD3^+^ T cells isolated from patient blood, whereas TCR-T therapies often utilize a mixture of both CD8^+^ and CD4^+^ T cells. CAR-T therapy entails the introduction of a chimeric antigen receptor gene, enabling the engineered T cells to recognize and eliminate tumor cells upon reinfusion. TCR-T therapy relies on the genetic transduction of TCR α/β heterodimers specific for tumor antigens, which enhances the affinity of TCR for MHC-antigen complexes. This modification allows T lymphocytes to efficiently recognize target cells through the physiological MHC-restricted antigen presentation pathway [164–167].

TCR-engineered T-cell therapy modifies autologous T cells to recognize and eliminate tumor cells through genetically introduced TCR genes. Following isolation from patients, T cells are transduced with defined TCR sequences via gene-editing techniques, enabling recognition of tumor-associated antigen–MHC complexes. This modification confers tumor antigen–specific recognition and cytotoxic capacity [168,169].

In CRC murine models, CAR-T cells engineered to secrete PD-1–TREM2 scFv (single-chain antibody fragment) demonstrated effective tumor eradication. The PD-1–TREM2 scFv suppressed PD-1/PD-L1 signaling and exhibited extended intratumoral persistence, indicating that PD-1–TREM2 scFv CAR-T-cell-based ACT represents a promising therapeutic avenue for CRC [170].

Karla et al. [171] designed a Dual-RevCAR T-cell strategy incorporating AND-gate logic, in which bifunctional adaptor molecules (RevTMs) selectively recognize carcinoembryonic antigen (CEA) and epithelial cell adhesion molecule (EpCAM). T-cell activation occurs only when both antigens are co-expressed within the same tumor cell, thereby enhancing therapeutic precision while minimizing off-target injury to normal tissues.

GCC19 CAR-T-cell therapy was assessed in a nonrandomized clinical trial involving patients with heavily pretreated mCRC. The study demonstrated acceptable safety and notable preliminary antitumor activity, yielding an objective remission rate of 40% and a median OS of 22.8 months. The therapeutic mechanism involves GCC-directed CAR-T cells augmented by CD19 CAR-T-cell expansion, which promotes proliferation and persistence of effector cells. This approach provided measurable clinical benefit and introduced a potential framework for extending CAR-T-cell therapy to solid tumors [172].

Ganjun Yu et al. [173] examined the combined therapeutic effect of low-dose decitabine with NY-ESO-1-specific TCR-T cells in MSS CRC. Decitabine converted the immunologically ‘cold’ tumor microenvironment into a more immunoreactive ‘hot’ state by upregulating NY-ESO-1 and other tumor-associated antigens, thereby enhancing the ability of TCR-T cells to specifically recognize and eliminate MSS CRC cells. Both *in vivo* and *ex vivo* analyses confirmed that this combination markedly increased tumor cell lysis and extended the survival of mice, highlighting its potential clinical application in MSS CRC therapy.

The details of the above research are as follows (Table 3).

**Table 3.** Staging, treatment, results, and conclusions of clinical/preclinical trials of ACT therapy for colorectal cancer

Name	Stages	Treatment	Result	Conclusion	References
PD-1-TREM2 scFv Secretory CAR-T cell therapy	Pre-clinical study	a. Anti-CEA CAR-T cells were successfully constructed and demonstrated the ability to autocrine the PD-1-TREM2 single chain variable fragment scFv antibody. b. Utilizing genetic engineering technology, the PD-1-TREM2 scFv antibody was integrated into CAR-T cells to enable them to target and specifically accumulate within the tumor microenvironment. c. In a mouse model of colorectal cancer, these modified CAR-T cells were administered intravenously to evaluate their antitumor effects.	a. The PD-1-TREM2 scFv antibody effectively inhibited the activation of the PD-1/PD-L1 pathway and reduced the proportion of MDSCs and TAMs. b. The modified CAR-T cells significantly enhanced effector T cell function and increased the infiltration and cytotoxic activity of CD8+ T cells within the tumour. c. Compared with conventional CAR-T cell therapy secreting PD-1 scFv alone, PD-1-TREM2 scFv-secreting CAR-T cells demonstrated stronger anti-tumour effects and more pronounced inhibition of tumour growth in the CRC mouse model.	The therapy is still in the preclinical research stage, and further investigation is needed to validate these findings.	[170]
Dual-RevCAR T-cell therapy	Pre-clinical study	a. A series of bifunctional adaptor molecules (RevTMs) specific for CEA and epithelial cell adhesion molecules (EpCAM) were developed, including scFv-based and IgG4-based anti-CEA RevTMs, as well as minibody-based and IgG1-based anti-EpCAM RevTMs. b. T cells expressing two distinct RevCARs, designated as one for signalling (SIG RevCAR) and the other for co-stimulation (COS RevCAR), which recognize CEA and EpCAM, respectively, were successfully engineered. c. The functionality of the Dual-RevCAR system was assessed through a chromium release assay, a luciferase-based killing assay, a cytokine release assay, and a murine tumour xenograft model to evaluate the specific killing capacity of T cells against CEA+EpCAM + colorectal cancer (CRC) cells.	a. *In vitro* experiments demonstrated that Dual-RevCAR T cells exhibited full activation exclusively in the concurrent presence of both CEA and EpCAM RevTMs, leading to specific killing of CEA+EpCAM + CRC cells along with concomitant cytokine release. b. *In vivo* experiments utilizing a mouse tumour xenograft model revealed that Dual-RevCAR T cells effectively suppressed tumour growth only upon simultaneous administration of both CEA and EpCAM RevTMs.	This approach enhances the safety and specificity of the treatment while minimizing potential damage to healthy tissues.	[171]
GCC19CAR-T cell therapy	Phase I Clinical Trial	a. Patients underwent lymphodepleting chemotherapy (fludarabine 30 mg/m² and cyclophosphamide 300 mg/m²) administered 3 days prior to GCC19CAR-T cell infusion. b. Patients were administered varying doses of GCC19CAR-T cells, with one cohort receiving 1 × 10⁶ cells/kg. c. Patients underwent periodic follow-up and were evaluated for safety.	a. Most patients experienced adverse reactions such as cytokine release syndrome and diarrhea, which were generally self-limited and controllable. b. Regarding efficacy, the overall response rate (ORR) was 40%, the median overall survival (OS) was 22.8 months, and the median progression-free survival (PFS) in the high-dose group was 6.0 months, which was significantly longer than the 1.9 months observed in the low-dose group.	Despite the limitations of a small sample size and short duration of objective remission, the results offer promising prospects and a foundation for further investigation of GCC19CAR-T cell therapy in MCRC and other solid tumors.	[172]
Low dose decitabine combined with NY-ESO-1 specific TCR-T cell therapy	Pre-clinical study	a. NY-ESO-1-specific TCR was constructed using genetic engineering techniques and transduced into T cells to generate NY-ESO-1-specific TCR-T cells. b. Colorectal cancer cell lines were treated with different concentrations of decitabine to quantify changes in the expression of NY-ESO-1 and other tumour-associated antigens. c. *In vitro* experiments: The specific cytotoxic activity of NY-ESO-1-specific TCR-T cells against tumour cells, along with the effect of decitabine pretreatment on TCR-T cell-mediated killing, was assessed using ELISPOT, LDH release assay and other methods. d. *In vivo* experiments: A xenograft mouse model was established. Mice received NY-ESO-1-specific TCR-T cells via tail vein injection, combined with decitabine treatment, to monitor tumour growth and mouse survival.	a. *In vitro* experiments: NY-ESO-1-specific TCR-T cells specifically recognized and lysed NY-ESO-1-expressing tumour cells, and decitabine treatment significantly enhanced the killing efficacy of TCR-T cells. b. *In vivo* experiments: The combination of decitabine and NY-ESO-1-specific TCR-T cell therapy significantly inhibited tumour growth and prolonged the survival of tumor-bearing mice.	The study demonstrated significant synergistic therapeutic effects, as evidenced by an increased tumor cell lysis rate and prolonged survival in xenograft models, indicating potential clinical applications.	[173]

### 3.4. Others

DC-derived exosomes are nanoscale, phospholipid membrane–bound vesicles that mediate intercellular communication and are under investigation as alternative platforms for cancer vaccines. These vesicles contain antigen-presenting components, including MHC class I, MHC class II, costimulatory molecules, and adhesion molecules, which activate antigen-specific cytotoxic T cells *in vivo* and suppress tumor growth. Combination of DC-derived exosomes with GM-CSF has been reported to trigger cytotoxic T-cell responses and enhance NK cell activity, while integration with ICIs further enhances antitumor T- and B-cell responses. Such approaches provide promising avenues for CRC therapy [174–176].

Bispecific antibodies (BsAbs) represent another immunotherapeutic modality designed to simultaneously recognize T cells and cancer cells, thereby inducing targeted cytotoxicity. Lei Wang et al. employed protein splicing technology to generate an IgG-like bispecific antibody (CD3 × EpCAM BsAb) capable of binding CD3 on T cells and EpCAM on tumor cells, enabling efficient T-cell redirection and activation. This construct demonstrated greater cytotoxicity *in vitro* compared with the parental EpCAM monoclonal antibody. Pharmacokinetic analysis revealed a half-life comparable to that of the maternal antibody, and *in vivo* evaluation confirmed antitumor efficacy in a SW480 xenograft mouse model. Collectively, these results indicate that CD3 × EpCAM BsAb holds considerable potential as a therapeutic candidate for CRC immunotherapy, offering a novel strategy for clinical intervention [177].

Reyes et al. [178] examined how heterogeneity and plasticity of CEA expression influence resistance to cibisatamab (CEA-TCB), a bispecific antibody targeting CEA, using patient-derived CRC organoids (PDOs). PDOs exhibited marked variability in CEA levels, with distinct CEA_hi_ and CEA_lo_ subpopulations. Antigen loss within CEA_lo_ cells contributed to acquired resistance, reflecting the plastic nature of CEA expression. RNA sequencing further demonstrated enhanced WNT/β-catenin signaling in CEA_lo_ cells, while pharmacologic inhibition of this pathway restored CEA expression and increased cibisatamab responsiveness. These observations indicate that CEA heterogeneity and plasticity may serve as predictive biomarkers for clinical outcomes and suggest that combining WNT/β-catenin pathway inhibitors with cibisatamab represents a rational therapeutic approach to improve CRC immunotherapy efficacy.

## 4. Discussion and conclusion

CRC, one of the most common malignancies of the digestive tract, demonstrates a strong link between disease progression, therapeutic response, and the dynamic nature of the TME. Intensive investigation of the TME has therefore become a central focus of contemporary CRC immunotherapy research. The present review delineates the dual roles of diverse immune and stromal cell populations within the CRC microenvironment, functioning either to promote or restrict tumor development. Recent progress in immunotherapy is summarized, including immune checkpoint inhibitors (anti-PD-1/PD-L1, CTLA-4, and the emerging target MS4A4A), cancer vaccines, and adoptive cell therapy. In addition, perspectives involving metabolic reprogramming, epigenetic regulation, and engineered cellular therapies are integrated to provide conceptual and strategic frameworks for overcoming immune suppression and advancing personalized therapeutic approaches.

The potential of immunotherapy to reshape CRC management is particularly evident in MSI-H/dMMR subgroups. Clinical translation requires comprehensive immune profiling to inform therapeutic choices, while the identification of predictive biomarkers—such as T-cell clonality, TLS density, and cytokine signatures—offers opportunities for refined patient stratification. Moreover, combining immunotherapy with conventional modalities may enhance therapeutic benefit and extend applicability to MSS tumors. A deeper understanding of the CRC immune landscape is expected to guide individualized strategies and ultimately improve long-term survival outcomes.

Our analysis reveals that DCs, TAMs, TANs, T cells, B cells, NK cells, CAFs, ECM, and EVs collectively contribute to the establishment of an immunosuppressive TME in CRC. Several mechanisms dominate this process: (I) TAMs and TANs adopt pro-tumor phenotypes (M2 and N2) under cytokine regulation such as TGF-β and CSF1; (II) T cell exhaustion arises from metabolic competition involving metabolites such as lactate and ammonia, together with the upregulation of immune checkpoints including PD-1 and TIGIT; (III) B cells exert dual roles, either promoting or suppressing immunity, with regulatory subsets such as LARS B driving immune evasion; (IV) CAF activation and ECM remodeling reinforce immune exclusion and metastatic dissemination; (V) EVs enable intercellular communication that reprograms immune populations toward immunosuppression.

In the therapeutic landscape, immunotherapy has transformed CRC management, particularly in MSI-H/dMMR tumors, yet treatment of MSS/pMR tumors remains challenging. Despite the remarkable success of immunotherapy in MSI-H/dMMR CRC, overcoming primary resistance in MSS/pMMR tumors remains a critical challenge. This resistance is largely attributable to a multifaceted immunosuppressive TME, characterized by low tumor mutational burden and neoantigen load, impaired antigen presentation, and dominant metabolic and cellular barriers [125,126]. The principal mechanisms underlying this immunosuppression can be elaborated as follows: (i) Metabolic competition, in which tumor cells outcompete T cells for glucose, resulting in lactate accumulation that suppresses CD8+ T-cell function through the MondoA–TXNIP axis while promoting Treg activity [54,70]; (ii) accumulation of immunosuppressive metabolites such as ammonia, which directly induces T-cell exhaustion [73]; (iii) upregulation of alternative immune checkpoints beyond PD-1 and CTLA-4, including LAG-3 and TIGIT [41,143]; and (iv) a dense stromal network composed mainly of CAFs and M2-type TAMs, which mediate both physical exclusion and chemical suppression of effector T cells [99,141].

To address these barriers, innovative combination therapeutic strategies are required. Recent clinical investigations have focused on combining ICIs with modalities that remodel the TME. For example, the combination of PD-1 inhibitors with anti-angiogenic agents such as bevacizumab can normalize tumor vasculature and enhance T-cell infiltration [127]. Epigenetic modulators—including histone deacetylase inhibitors like chidamide—have demonstrated potential in reversing immune suppression. Notably, a triple regimen comprising sintilimab, chidamide, and bevacizumab has shown promising efficacy in MSS/pMMR CRC [140]. Similarly, targeting specific TME components, such as MS4A4A on TAMs to reprogram them toward a pro-inflammatory phenotype, can act synergistically with PD-1 blockade to reinstate antitumor immunity [141]. The exploration of LAG-3 inhibition in combination with PD-1 blockade represents another promising strategy to mitigate T-cell exhaustion [132,143]. Furthermore, modulating T-cell metabolic fitness—for instance, by using succinate to enhance stemness and mitochondrial function [65] or inhibiting the MondoA–TXNIP axis to alleviate lactate-induced suppression [70]—has emerged as a novel frontier for improving ICI efficacy.

These multifaceted approaches underscore an essential future direction: shifting from single-agent ICI therapy toward personalized, multi-target regimens that concurrently disrupt the metabolic, cellular, and checkpoint barriers within the MSS/pMMR TME. Combination regimens integrating ICIs with anti-angiogenic agents, chemotherapy, or emerging checkpoint targets such as LAG-3 demonstrate potential to convert immunologically ‘cold’ tumors into ‘hot’ ones. Additionally, personalized neoantigen vaccines, CAR-T/TCR-T strategies, and bispecific antibodies represent advancing modalities with considerable translational promise.

However, several limitations remain in current research. Mechanistic understanding largely relies on preclinical systems, including murine CRC models and cell lines, which only partially reflect the heterogeneity and complexity of human tumors. Translational applicability is further restricted by the absence of reliable biomarkers capable of predicting patient responses to immunotherapy. Most clinical investigations to date consist of early-phase trials with small cohorts, underscoring the necessity for larger randomized controlled studies to substantiate preliminary observations.

Future directions should emphasize a more integrated analysis of the CRC immune microenvironment, with particular attention to intercellular communication among immune subsets and their dynamic interactions with tumor cells. Therapeutic opportunities may emerge from targeting intrinsic T-cell metabolic pathways. Approaches such as succinate supplementation to enhance T-cell stemness and mitochondrial function [66], the application of ferroptosis inducers [66], or inhibition of the MondoA–TXNIP axis to counteract lactate-driven suppression [70] hold potential to mitigate immune dysfunction and improve immunotherapy outcomes in CRC.

Advancing immunotherapy for CRC requires coordinated multidisciplinary efforts, individualized treatment designs, and innovative therapeutic strategies. High-resolution analysis of TME dynamics through integrative multi-omics approaches—including single-cell RNA sequencing, spatial transcriptomics, and proteomics—combined with novel therapeutic regimens such as dual checkpoint blockade with metabolic modulators, represents a promising avenue to overcome resistance in MSS/pMMR CRC. The establishment of patient-derived organoids and humanized mouse models further strengthens the predictive capacity of preclinical research and supports the discovery of new therapeutic targets. Continued technological progress and refined insights into the CRC immune microenvironment are expected to drive the development of next-generation immunotherapies, expanding the range and efficacy of treatment options for CRC patients.

## Data Availability

Data sharing is not applicable to this article as no data were created or analysed in this study.
